# Nanoscale structural defects in oblique Ar^+^ sputtered Si(111) surfaces

**DOI:** 10.1038/s41598-019-52099-4

**Published:** 2019-10-29

**Authors:** Divya Gupta, Mahak Chawla, Rahul Singhal, Sanjeev Aggarwal

**Affiliations:** 1 Department of Physics, Kurukshetra University, Kurukshetra, 136119 India; 2 Malviya National Institute of Technology, Jaipur, Rajasthan 302017 India

**Keywords:** Materials science, Nanoscience and technology

## Abstract

The present endeavor investigates the controlled surface modifications and evolution of self-assembled nano-dimensional defects on oblique Ar^+^ sputtered Si(111) surfaces which are important substrates for surface reconstruction. The defect formation started at off-normal incidences of 50° and then deflates into defined defect zones with decrease in oblique incidence, depending strongly on angle of ion incidence. Interestingly, it is observed that mean size & height decreases while average density of these defects increases with decreasing oblique incidence. Non-linear response of roughness of irradiated Si(111) with respect to oblique incidence is observed. Crystalline (c-Si) to amorphous (a-Si) phase transition under oblique argon ion irradiation has been revealed by Raman spectroscopy. Our analysis, thus, shows that high dose argon ion irradiation generates of self-assembled nano-scale defects and surface vacancies & their possible clustering into extended defect zones. Explicitly, ion beam-stimulated mass transport inside the amorphous layers governs the observed defect evolution. This investigation of crystalline (c-Si) coupled with amorphous (a-Si) phases of nano-structured surfaces provides insight into the potential applications in the nano-electronic and optoelectronic devices thus, initiating a new era for fabricating multitude of novel structures.

## Introduction

In integrated circuit technology, silicon is nowadays exploited as a potential candidate for the production of various nano-scale applications like optoelectronic devices, IR detectors, and FET’s^1^. Although there are several approaches for fabricating such devices, but ion implantation is the best suited technique as it induces the formation of buried amorphous layers with precise interfaces to accentuate different properties of silicon, hence, intensifying possibility of fabricating novel structures^1–3^. The augmented interest in VLSI technology, in turn, brings the usage of ion implantation, predominantly in low energy range, to the forefront^3,4^. This low energy implantation, however, results in severe alterations in the subsurface region of the target matrix crucially depending on the implantation conditions like energy, implantation dose and angle of the incoming ion^2,3^. Thus, for keV and MeV ion implantation to be key engineering tool in the development of semiconductor industry^4^, it becomes necessary to address the issue of surface modifications and the factors governing these alterations^1,3^.

During the interaction of ion beam with target medium, the incident ions suffers a number of discrete collisions and loses its complete energy in nuclear and electronic collisions in the target material^1,5^. These ions get finally deposited over the depth R_P_ called as projected range, dependent on the irradiation parameters. Nuclear energy loss in comparison to electronic energy loss is found to be majorly responsible for the surface modifications in the target matrix at low energies typically of few keV’s. This energy loss due to elastic collisions results in defects and strains leading to surface and near surface alterations in the target matrix^1,5,6^. The resulted defect zones are irregularities in the target material having large clustering of interstitial atoms and/or vacancies^1,5^.

The potential applicability and reliability of Si in the nano-device fabrication is directly related to its surface characteristics^1,2^. The implantation induced defects and strains generally inhibit carrier mobility and enhance leakage current while induced surface roughness crucially affects the performance of these Si based devices^6,7^, hence, it becomes important to characterize these defects along with induced surface roughening and govern the controlling parameters. In addition, the fabrication of the self-assembled and densely packed surface nano-structures, owing to ion implantation at oblique incidences, are also much in limelight as these surface structures can be better used for controlled production of group IV nano-scale devices in analogy to self-organized growths^7,8^.

The previous studies stated in the literature discusses the ion implantation induced amorphization and defects over Si(100), Si(110) and Si(001) surfaces by high energy ion implantations where electronic energy loss process dominates, such as 1.5 MeV Sb ions^1^, 1.4 MeV Sb implantation^9^, 3 MeV B^+^ and Si^4+^ implantation^10^, 1 MeV Sn implantation^11^, 8.3 MeV Si implantation^12^ or at low irradiation fluences^13,14^. Moreover, using low energy ion beam sputtering, many researchers^15–19^ reported the evolution of ripples and nanodots on these semiconductor surfaces instead of the formation of surface defects. Further, the role of sputtering in amorphization and defect formation under keV ion implantation remains poorly understood, particularly in Si(111) substrates. These Si(111) substrates are key engineering substrates for surface reconstruction and high oxidation rate as it has highest atomic density in comparison to other planar orientation of Si. Infact, there are no studies in the existing literature addressing high irradiation flux and simultaneous sputtering induced nanoscale defects over Si(111) surfaces highlighting the role of oblique incidences under keV energy range. This, in turn, motivated us to investigate the role of oblique incidences in surface structuring over Si(111) surfaces due to Ar ion sputtering, which are pivotal substrates for surface reconstruction. In this regard, present studies report the evolution of nano-dimensional defects in Si(111) under oblique Ar^+^ sputtering. This nano-scale defect growth that appears on irradiated surfaces strongly depends on the oblique incidence. The parameters related to nanoscale defect structures and induced root-mean-square (rms) surface roughness in irradiated surfaces has been evaluated as a function of oblique incidence. Raman-modes reveal the crystalline to amorphous phase transition under argon ion sputtering. As a consequence, surface behavior becomes multifaceted with both induced roughness and stress varying as a function of off-normal incidence of bombarding ions. Our experimental results reveal that ion beam stimulated mass rearrangement in the amorphous layers leads to angle dependent alterations in the surface structure of the irradiated Si(111) samples. Thus, ion beam sputtering leads to the evolution of self-assembled and densely packed defect zones on the Si(111) surfaces under high flux irradiation.

## Experimental Details

In the present work, single side polished p type-Si(111) substrates of size 2 × 2 cm^2^ have been used. These samples were sputtered with Ar^+^ ion beam having energy of 80 keV to a dose of 1 × 10^17^ Ar^+^cm^−2^ at oblique incidences of 30°, 40° and 50° using 200 kV Ion Accelerator available at Ion Beam Center, Department of Physics, Kurukshetra University, Kurukshetra.

The morphological changes in the Si(111) surfaces after irradiation were examined in Scan-Asyst HR mode by Nanoscope V Multimode-8 system. The parameters of surface nano structures such as average size, height and density were computed using the Nanoscope 1.8 software provided with the AFM. Particularly, the discrete fast Fourier transformation (FFT) and Power Spectral density (PSD) were taken to quantify the lateral correlation of the self-assembled structures. For the profound evaluation of the surface roughness variation of the evolved surfaces, rms (root mean square) roughness has been evaluated^20–22^. To analyze the structural changes in irradiated Si(111) samples, Raman spectrometry has been employed. The Raman plots were obtained by STR 500 confocal micro-Raman spectrometer with diode pumped solid state laser having excitation wavelength of 532 nm in backscattering geometry.

## Results and Discussion

### Morphological characterization of Ar^+^ sputtered Si(111) surfaces

**Figure 1:**
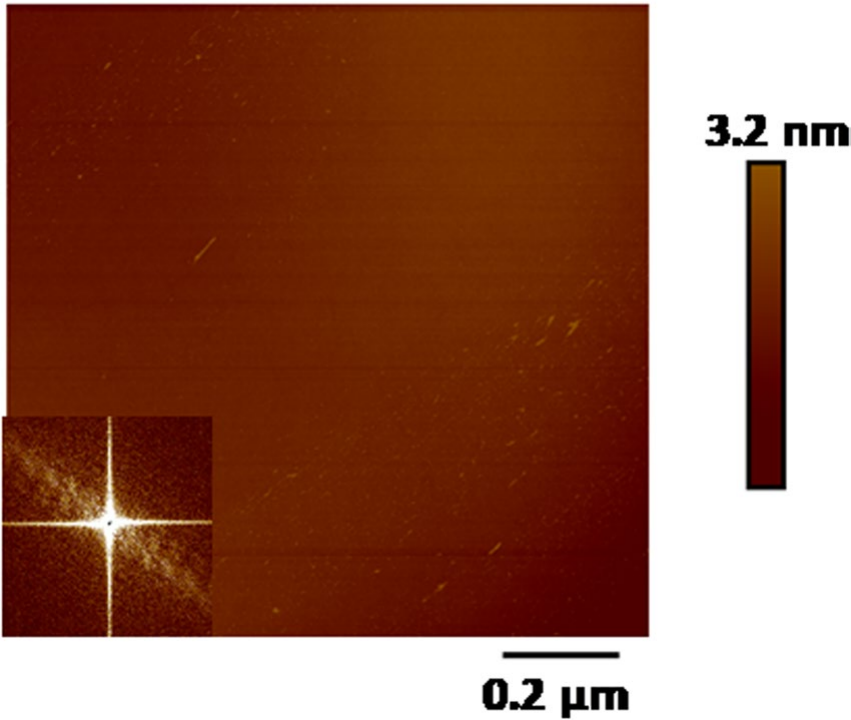
AFM micrograph of surface morphology of un-irradiated Si(111).

Figure 1 presents the 1 × 1µm^2^ AFM topographic image of Si(111) surface before irradiation, where smoothness over the surface can be observed. The corresponding inset presents the 2D Fast Fourier transform (FFT) image. Its FFT image confirms the smoothness as there is no lateral correlation among different points over the sample surface. Further, we have quantified the roughness of the sputtered Si(111) surfaces and are summarized in Table 1. For the un-irradiated surfaces, roughness is found to be 0.77 ± 0.04 nm.

**Table 1:** Size, height and density distribution of nanoscale defects & roughness of Si(111) surfaces as a function of oblique incidence.

Specimen	Size (nm)	Height (nm)	Density (cm^−2^)	Roughness (nm)
Un-irradiated	—	—	—	0.77 ± 0.04
Si(111) irradiated at 50°	87 ± 06	12 ± 04	4.5 × 10^8^	4.36 ± 0.21
Si(111) irradiated at 40°	54 ± 04	08 ± 02	9 × 10^8^	2.32 ± 0.12
Si(111) irradiated at 30°	42 ± 05	05 ± 01	11 × 10^8^	1.78 ± 0.09

**Figure 2:**
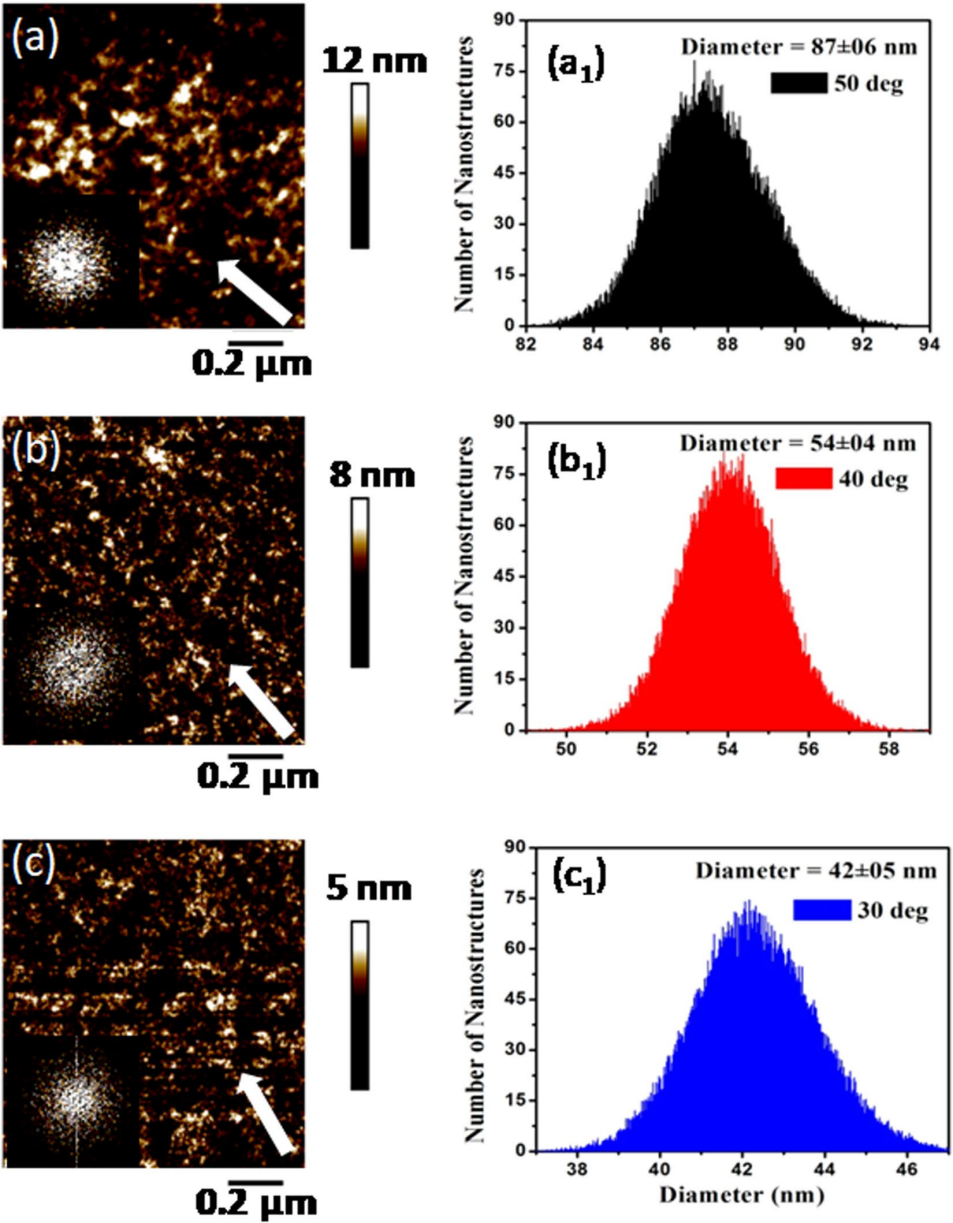
AFM micrographs of surface morphology of Si(111) exposed to 80 keV argon ions for a fluence of 1 × 10^17^ Ar^+^cm^−2^ at off-normal incidences of (**a**) 50°, (**b**) 40° and (**c**) 30°. The size distribution of nano-structures for the off-normal incidences of (**a**_**1**_) 50°, (**b**_**1**_) 40° and (**c**_**1**_) 30°.

The AFM micrographs in Fig. 2(a–c) show the Si(111) surface modification and morphological evolution after 80 keV argon ion sputtering at oblique incidences of 50°, 40° and 30°. The 2D Fast Fourier transforms (FFT) images corresponding to these AFM micrographs are presented in the insets of Fig. 2(a–c) respectively. Interestingly, in contrast to Fig. 1 (before irradiation), surface morphologies of sputtered Si(111) surfaces in Fig. 2 after irradiation reveals the signature of nano-scale structural defects. We have evaluated the size, height and density of these nanoscale defects depending upon the angle of argon beam incidence along with surface roughness and the results are tabulated in Table 1.

Figure 2(a) shows the AFM micrograph for 80 keV Ar^+^ sputtered Si(111) surface at oblique incidence of 50°. A multitude of nano-dimensional defects can be observed on the irradiated Si(111) surface. Formation of such defects is attributed to the surface disorder created by the obliquely incident argon ion beam. For oblique incidence of 50°, most of the nanostructures have diameter 87 ± 06 nm and height of 12 nm Fig. 2(a,a_1_). The density of these nanoscale defects comes out to be 4.5 × 10^8^ cm^−2^ which is surprisingly much lesser than irradiated argon ion fluence. The corresponding FFT image shows the random ordering among the evolved nanostructures resulting in increased roughening of the surface to a value of 4.36 ± 0.21 nm at this stage of irradiation.

**Figure 3:**
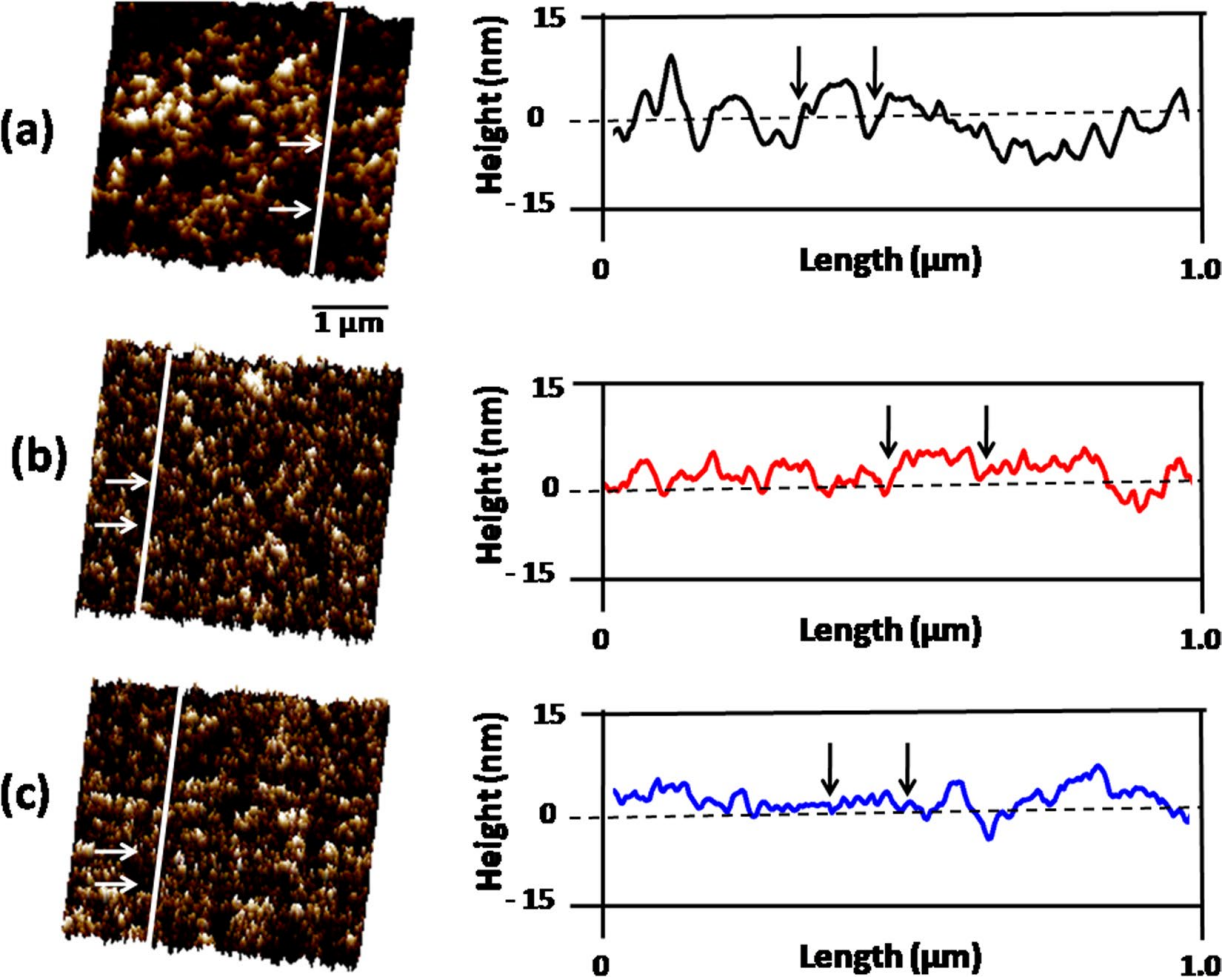
3D AFM images and AFM-section analysis of surface morphology of Si(111) exposed to 80 keV Ar^+^ ions at oblique incidences of (**a**) 50°, (**b**) 40° and (**c**) 30°. The arrows mark the size of evolved defect.

On irradiation at the smaller oblique incidence of 40°, the nanostructures have become smaller in size. Figure 2(b) displays the fragmentation of bigger sized defects into smaller defects Fig. 2(b,b_1_). As a consequence, the diameter of most of the nanostructures has reduced to 54 ± 04 nm and height to 8 nm (Fig. 3). At this stage, an abrupt enhancement in the density of evolved nano-dimensional defects occurs. The density of nanostructures is about 9 × 10^8^ cm^−2^ for oblique incidence of 40°. Infact, a drastic decrease of the roughness of irradiated Si(111) surface at this off-normal incidence has been seen. The FFT image in inset depicts the weak short range ordering of these nanoscale defects.

With further decrease in irradiation angle to 30°, AFM image (Fig. 2(c)) reveal that the fragmentation of these defects still continues. This causes further decrease in the mean size to 42 ± 05 nm and height to 5 nm of these defects Fig. 2(c,c_1_). also, density of these nanostructures increases to about 11 × 10^8^ cm^−2^. Interestingly, the FFT image (shown in lower inset) depicts the square ordering of these nano-defects indicating significant decrease in rms roughness of irradiated surfaces.

From Table 1, we deduce that roughness exhibits decreasing behavior as a function of oblique incidence. The least roughness observed at oblique incidence of 30° is due to predominant diffusion mechanism induced due to enhanced amorphization at this stage. The corresponding decrease in roughness as a function of oblique incidence point towards ordering of nanoscale defects evolved on the surfaces of irradiated specimens (Fig. 2). Hence, it can be inferred that square ordering of nano-defects from 50° to 30° is accompanied by the smoothening of the sputtered Si(111) surfaces.

Most importantly, estimations of defect density for all the oblique incidences reveal that these values are much lower than that of the irradiation fluence. The lower defect density can be explained in the light of Gibbons^23^ model for defect evolution upon irradiation. Figure 3 shows the 3D images and AFM-section analysis of 1 × 1 µm^2^ micrographs of the Si(111) surfaces for oblique incidences of (a) 50°, (b) 40° and (c) 30°.

Figure 3(a) shows a 1 × 1 µm^2^ AFM image of Si(111) surface for oblique incidence of 50° and its section analysis. Here, defect is 80 nm and 10 nm in lateral & vertical dimensions. But there are some defects of smaller size and height as well. Figure 3(b) presents a 1 × 1 µm^2^ 3D AFM image of Si(111) surface for oblique incidence of 40°. At this stage, surface displays both small and big size defects. Defect is found to be 55 nm in lateral and 7 nm in vertical direction as estimated from its section analysis and this bigger defect comprises several smaller defects. Further, smaller defects of 18 nm in linear dimensions have been noticed using section analysis. Further, several big defects are partly covering small defects. Similar behavior is observed in Fig. 3(c) for the oblique incidence of 30°. Again, section analysis reveals defect of size 40 nm and height of 4 nm. In addition, both small as well as big defects can be easily seen. Section analysis of these smaller defects reveals dimensions of 13 nm in lateral direction. These micrographs clearly depict that the larger defects are overlapped by several smaller defects for all oblique incidences. Hence, we tried to recalculate the defect density and found it to be 0.5 × 10^13^ cm^−2^.

According to Gibbons model, amorphous layers are a result of single or multiple collision cascades between the incoming ion and target atoms^23,24^. Hence, using this approach,

1 $$\frac{{A}_{A}}{{A}_{0}}=1-{e}^{-{A}_{1}\varphi }\mathop{\sum }\limits_{k=0}^{m}\frac{{A}_{1}{\varphi }^{k}}{k!}$$

Here, ${A}_{1}$ is the surface area corresponding to single impact ion, *k* is the summation parameter, $\phi$ is the fluence and m is the overlapping number in Eq. 1. For given ion fluence, we calculated overlapping parameter and found m = 3, i.e. about 3 ions contribute to single defect formation. This, in turn, accounts for the deviation in the defect density from the argon ion beam fluence.

### Structural characterization of oblique Ar^+^ sputtered Si(111) surfaces

Raman scattering is a potential characterization technique for the distinction of crystalline and amorphous phases in semiconductors by identifying their Raman active lattice modes^25–28^. For silicon, the Raman spectrum is primarily characterized by scattering through Longitudinal Optical (LO) vibrations and Transverse Optical (TO) phonons vibrations corresponding to Brillouin zone center^4,29^. Interestingly for (111) face, scattering by both these modes of brillouin zone center are feasible. Keeping this in mind, we have explored the structural modifications associated with these LO and TO modes after argon ion irradiation at various oblique incidences^4,28,29^. For Diode pumped laser having incident wavelength of 532 nm, the Raman probing depths are nearly 770 nm and 100 nm in crystalline and amorphous phases of Si(111), respectively^28,29^. Thus, in our case, Raman measurements are majorly dealing with surface and near surface region modifications.

**Figure 4:**
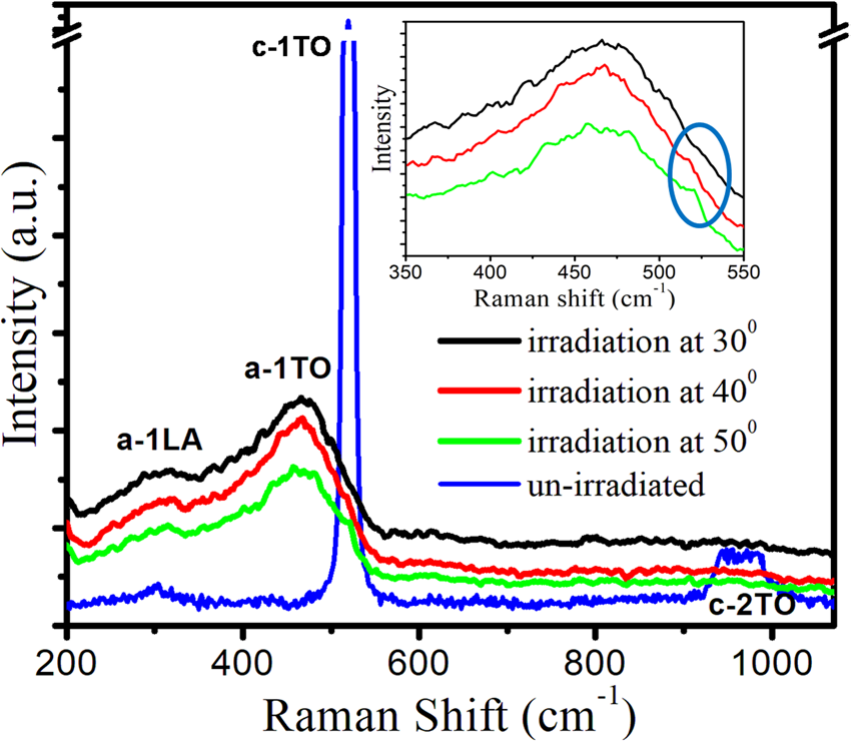
Raman spectra of un-irradiated and 80 keV Ar^+^ sputtered Si(111) samples for various oblique incidences.

Figure 4 displays the Raman spectra of un-irradiated and 80 keV Ar^+^ sputtered Si(111) specimens as a function of oblique incidence. All the 80 keV Ar^+^ sputtered spectra have been moved vertically for clarity but the intensity scale is same.

In case of un-irradiated sample, a narrow first order peak at 520 cm^−1^ corresponding to optical phonon mode (1TO) of crystalline silicon (c-Si) and a broad second order peak (2TO) centered at 975 cm^−1^ in the range of 930 cm^−1^–1030 cm^−1^ has been observed^25–32^. After irradiation at different oblique incidences of 50° to 30°, the spectra consist of an amorphous phase (a-Si), being characterized by a broad band centered at (470 ± 4) cm^−1^ due to transverse optic TO mode. The appearance of this amorphous broad band in the Raman spectra of irradiated specimens signifies the damage induced amorphization. A weaker band at 300 cm^−1^ appears and corresponds to brillouin zone edge LA vibrations^25–28^. It is evident that argon ion irradiation results in the enlargement of the amorphous regions at the cost of the crystalline content.

**Figure 5:**
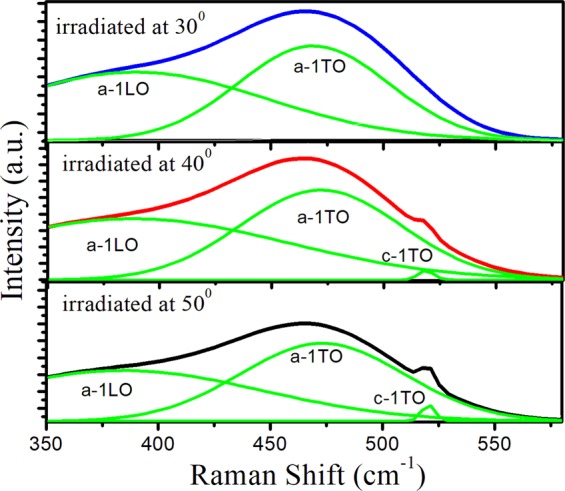
Deconvoluted Raman spectra of 80 keV Ar^+^ sputtered Si(111) at oblique incidences of 30°, 40° and 50°.

In addition, brillouin zone edge LO vibrations around 400 cm^−1^ seem to have merged with the a−1TO peak. We have plotted the region 350 cm^−1^ to 550 cm^−1^ to assess the contribution from crystalline peak (a-Si) in the inset of Fig. 4. After its deconvolution in Fig. 5, the c-1TO, a-1TO and a-1LO peaks^25–27^ are separated in the spectra for oblique incidences of 50° and 40° while for oblique incidence of 30°, only a-1TO and a-1LO peaks are separated (Fig. 5). Parameters such as center, width and intensity of the deconvoluted a-1TO, c-1TO and a-1LO peaks have been calculated and are summarized in Table 2.

**Table 2:** Center and width of a-Si, c-Si and a-1LO peaks for un-irradiated and irradiated specimens.

Specimen	Center of a-1TO peak (cm^−1^)	Center of c-1TO peak (cm^−1^)	Center of a-1LO peak (cm^−1^)	Width of a-1TO (cm^−1^)	Width of c-1TO (cm^−1^)	Width of a-1LO (cm^−1^)
Un-irradiated	—	520	—	—	04	—
Si(111) irradiated at 50°	470	519	390	65	08	112
Si(111) irradiated at 40°	468	518	389	64	06	110
Si(111) irradiated at 30°	466	—	386	62	—	107

It is clear from Fig. 5 and Table 2 that the oblique Ar^+^ sputtering results in the enlargement of amorphous region at the expense of crystalline phase. Further, it can be inferred that with decrease in oblique incidences from 50° to 30°, the peak position of a-1TO, a-1LO shifts to lower wavenumber and exhibits asymmetric FWHM with lower wavenumber. Full width at half maxima (FWHM) of Raman peak principally reflects the defect density. For obliquely argon ion irradiated Si(111) at different oblique incidences of 50°, 40° and 30°, decrease in linewidth of Raman 1-TO peak with decrease in oblique incidence (as clear from Table 2). clearly points towards the decrease in generation of point defects.

Furthermore, the presence of small contribution of crystalline phase near 520 cm^−1^ for argon sputtered Si(111) at oblique incidences of 50° and 40° samples is not due to underlying c-Si lattice but it may be due to the presence of small crystalline pockets in the vicinity of amorphous zones, available after the irradiation at 50° and 40° resulting in this Raman peak at 519 and 518 cm^−1^ having width of 8 and 6 cm^−1^. This observed Raman peak shift towards lower wave number may be accounted in terms of phonon and quantum confinement effect^9,13,25^. In case of crystalline Si, only the optical phonons close to the zone center were contributing in the Raman Spectrum due to periodicity of the crystal lattice. But due to argon ion sputtering, this periodicity of the crystal lattice is broken and phonons away from the Brillouin zone center starts contributing in addition to phonons close to zone center. As a result, asymmetric peak at lower wavenumber is observed in comparison to crystalline peak^9,25^.

Surprisingly, at oblique incidence of 30°, complete transformation from crystalline to amorphous phase has been observed. This is confirmed by the absence of crystalline content for this oblique incidence (Table 2). Hence argon ion sputtering at oblique incidence of 30° for a fluence of 1 × 10^17^ ions cm^−2^ induces crystalline to amorphous (c/a) phase transition in Si(111). Lavrentiev *et al*.^30^ reported the c/a phase transformation in Si(111) under 3.035 MeV Au^+^ implantation with a fluence greater than 5 × 10^14^ ions cm^−2^ under normal incidences.

**Figure 6:**
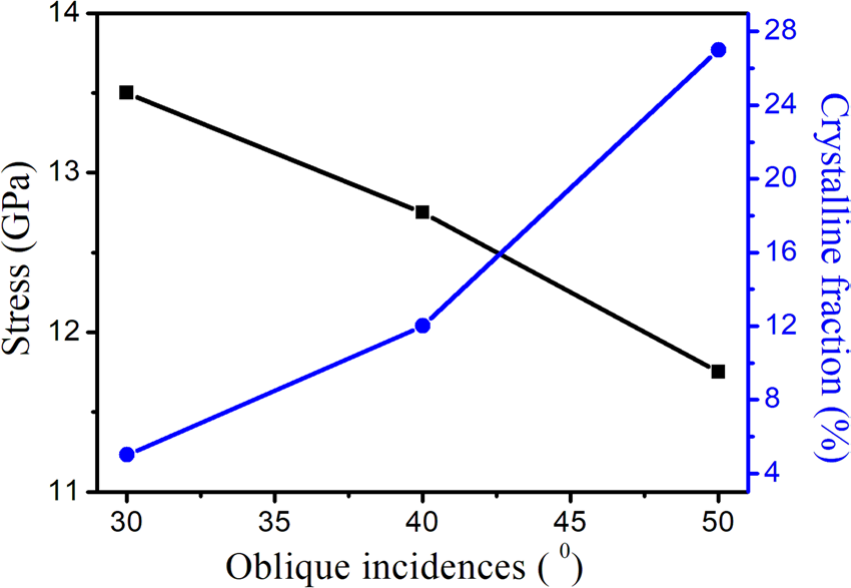
Variation in stress and crystalline fraction for different oblique incidences.

Hence, structural measurements demonstrate red shift in Raman peak position and simultaneous line-width narrowing with decrease in oblique incidence. This surprising result is attributed to oblique incidence induced Ar^+^ sputtering which causes the erosion of the Si(111) surface atoms and as a consequence leads to line-width narrowing of these peaks. However, Sahu *et al*.^25^ observed red shift in Raman peak position along with line width broadening with increase in implantation fluence. Also, Mishra *et al*.^29^ showed that with increase in ion fluence, the position of a-Si peak shifts to lower wave number and consequent FWHM broadening. Dey^9^ *et al*. have demonstrated red shift in Raman peak position and correlated it with the production of stress. Furthermore, for quantification of degree of amorphization, the crystalline fraction (X_C_) in the irradiated Si(111) has been evaluated^28^ and the results are presented in Fig. 6.

Interestingly, argon ion irradiation induced stress in the irradiated Si(111) results in the shifting of characteristic Raman peak^25,28,29^. We have estimated the induced stress for different off-normal incidences from the shift of the Raman phonon mode present at 521 cm^−1^ ^28,29^ using the relation

2 $$\sigma (MPa)=250\times \Delta \omega (c{m}^{-1})$$

Here, σ is the amount of the stress $\Delta \omega$ is the peak shift. The calculated stress for different oblique incidences has been plotted in Fig. 6.

Figure 6 shows that the contribution of crystalline (c-Si) peak is maximum (26%) for oblique incidences of 50° and gradually decreases with the decrease in oblique incidence. Thus, crystalline fraction X_c_ in irradiated Si(111) vanishes for lowest oblique incidences of 30°. Hence, crystalline to amorphous (c/a) transition occurs as a result of oblique Ar^+^ sputtering under high flux. This reduction in crystallinity suggests the presence of point defects and their random distribution in the lattice^28,29^. Thus, we can conclude that crystalline areas are embedded in the amorphized-defected zones and the amount of structural modification occurring in the Si lattice depends crucially on the angle of incoming argon ions. Similar studies on calculation of implantation induced stress and crystalline fraction have been reported in the literature^9,13,25,28,29^. Dey^9,13^ *et al*. has observed a significant increase in the stress for 1.5 MeV Sb^+^ implanted Si(100) with corresponding increase in ion dose. Sahu *et al*.^25^ calculated the stress present in the Si(100) system due to Au^+^ implantation at different fluences. They observed increase in tensile stress with ion fluence and accounted in terms of phonon confinement effect. Further, Mishra *et al*.^29^ showed that with increase in ion fluence, the crystalline volume fraction decreases and becomes zero for 5 × 10^15^ Co^+^ in Si(100) symbolizing crystalline to amorphous phase transition.

Further, it can be inferred that the stress in irradiated Si(111) samples increases with decrease in oblique incidence. The induced stress due to the softening of longitudinal and transverse optical modes reduces strain in the irradiated samples and hence leads to smoothening of the surfaces (as observed using AFM measurements).

In crystalline Si, tetrahedral bond angle between Si-Si bonds is 109°28”. Argon ion irradiation weakens the covalent bonds of the Si(111) resulting in amorphous system consisting of distributed bond angles^25–29^. The variation in FWHM of amorphous TO peak for irradiated Si(111) from crystalline Si gives qualitative analysis of amount of disorder and measures lateral ordering. The bond angle deviation ($\varDelta \theta$) in the irradiated samples has been computed using the relation^29^

3 $$FWH{M}_{TO}=15+6\Delta \theta $$

**Figure 7:**
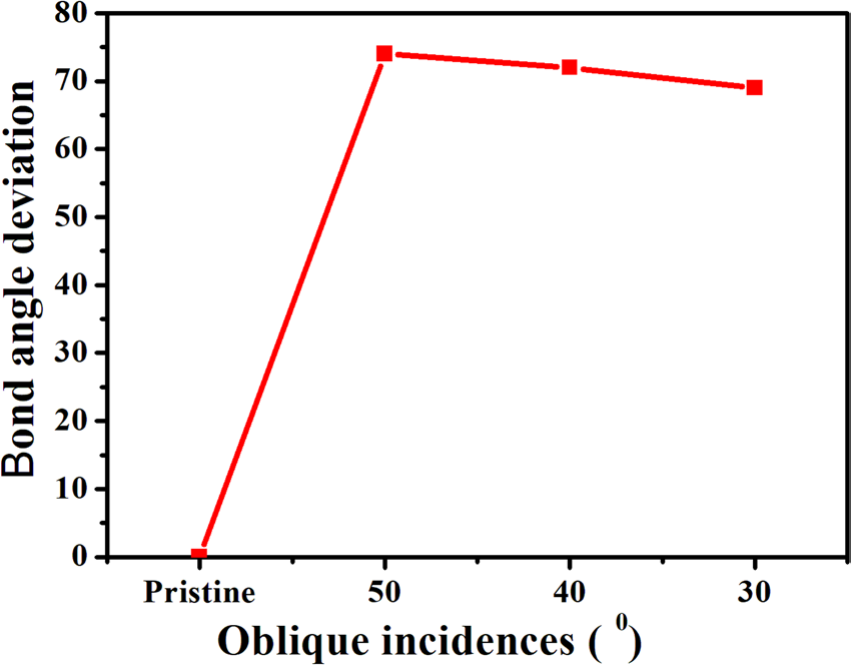
Bond angle deviation for different oblique incidences.

The calculated deviation in Bond angle with incidence angle is displayed in Fig. 7.

It can be inferred that for a-Si, the bond angle differs by 5° with decrease in oblique incidence from 50° to 30° (Fig. 7). The decrease in bond angle with oblique incidences is a clear indication of short range (SR) structural order in the amorphous region. Bond angle variation in the range 0° < $\varDelta \theta$ < 7° is in the transition regime of amorphous to crystalline phase^29^. Thus, irradiation at higher oblique incidence produces small amount of amorphous phase in a crystalline matrix while decreasing the off-normal incidence leads to enhanced amorphization with decrease in bond angle deviation. The increase in amorphization with decrease in oblique incidences goes in line with deconvolution results. Literature reports such calculations on bond angle deviation^9,26–29^. Dey^9^ *et al*. have calculated bond angle deviation for Sb^+^ implanted Si(100). Mishra^29^ *et al*. reported that bond angle deviation increases from 3° (for a fluence of 5 × 10^13^ ions cm^−2^) to 12.2° (for a fluence of 5 × 10^15^ ions cm^−2^) and correlated with presence of crystalline and amorphous phases.

Thus, the interplay between defect generation and damage induced amorphization leads to angle dependent alterations in the surface structure of the irradiated Si(111) samples as evident from Raman studies.

As far as creation of defects near the surface is concerned, our results reveal that for oblique incidences, the defects are formed closer to the surface for keV energy range and high flux of irradiation as confirmed by Raman measurements. But for normal incidences, defects are generally produced in the near surface regions by MeV beams^9,13^ which are not so convenient to use.

### Power Spectral Density (PSD) approach for quantification of underlying mechanism

Roughness of the un-irradiated and irradiated Si(111) surfaces has been quantified in terms of root-mean-square roughness which is a statistical parameter involving only the vertical distribution of roughness i.e. z-axis. Moreover, such measurement of roughness depends on scanning methods like scanning scale, scanning speed etc. Hence, for the profound evaluation of both lateral distribution (x and y), we have used Power spectral density (PSD) approach as it provides both lateral and vertical signals captured from AFM images.

Additionally, Power spectral density (PSD) approach provides statistical information regarding mechanisms governing the surface nanostructuring. PSD spectra have been used to evaluate the nanostructuring dynamics and to elucidate the underlying mechanism as a function of the oblique incidence^33,34^.

**Figure 8:**
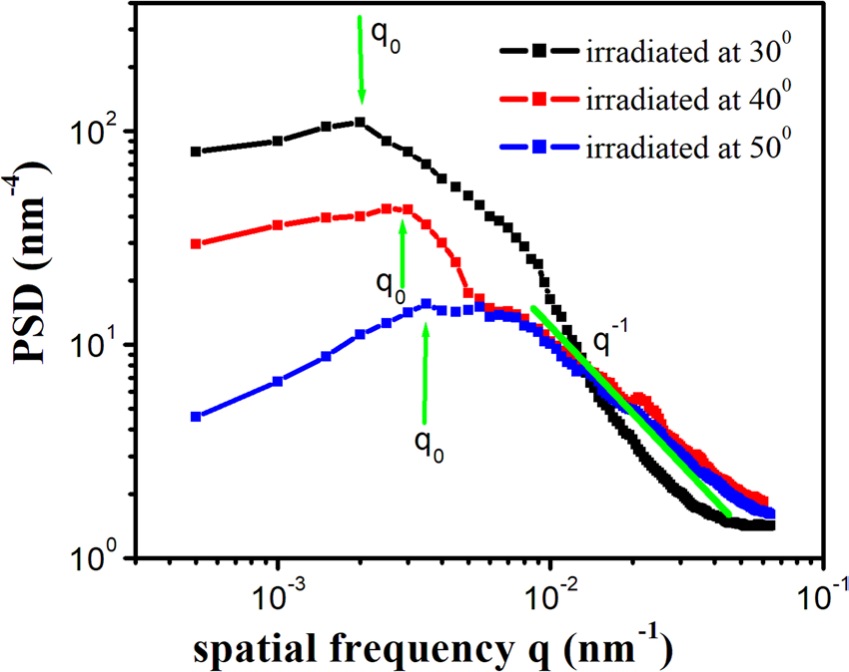
Log-Log plot of PSD as a function of spatial frequency.

We have obtained the PSD functions from Fourier transform (FT) of irradiated Si(111) surface using Nanoscope 1.8 software^33^. Figure 8 presents the log-log plot of 2D-PSD spectra as a function of frequency for 80 keV Ar^+^-sputtered Si(111) samples for off-normal incidences of 30°, 40° and 50°.

Broadly, PSD curve of a surface can be decomposed into two distinct regions of relevance governed by surface corrugations where, corrugation refers to the slope of a line relating two arbitrary points over the surface. One is the behavior at small q, (correlation length η (=l/q)) which governs the lateral size of the surface features and the second is the response at large q, which describes the nature of the roughness. In this way, the corrugation becomes small for q < q_0_ i.e. points separated by a length larger than the correlation length. Consequently, in this region, the surface remains flat. Thus, we can assume the PSD function to be independent of q (with the height value q_0_), while these surface corrugations become significant for length scale larger than correlation length. As a result, PSD should decrease for q > qo and a frequency dependent PSD function is expected in this region^22,33,34^.

Figure 8 reveals that this response of PSD function is observed for different oblique incidences of 50°, 40° and 30°. Moreover, the positions of q_0_ are marked for different oblique incidences in Fig. 8. It is clear that correlation length η (=l/q) increases with decrease in oblique incidence. This evidences the fragmentation of the bigger defects into smaller ones as observed in the AFM micrographs (Fig. 2). Further, for larger values of q, nature of roughness can be inferred from the Fig. 8. The value of q_0_ is highest for oblique incidence of 50° depicting that sputtering process leads to much rough surfaces at this oblique incidence. Interestingly, the value of q_0_ shifts towards lower values with corresponding decrease in oblique incidence. This, in turn, reveals the smoothening of the surfaces with decrease in oblique incidences in analogy with the rms roughness measurements. Hence, PSD analysis quantifies the nature of un-irradiated and irradiated surfaces and confirms the rms roughness measurements.

Further, the frequency dependent behavior of PSD function is given as^34^

4 $$PSD=A{q}^{-b}$$

Here, b is a real number and A is the proportionality constant. Using the power law fitting of the PSD spectra at high frequencies, the values of b have been calculated. The calculated value of b is marked in Fig. 8. The observed slope of the PSD function for q > q_0_ is ${q}^{-1}$ for all the oblique incidences and is characteristic behavior of surface confined viscous flow process^33–37^. This process leads to the decrease in the size and height distribution of nano-defects as a function of oblique incidences. Furthermore, decrease of the intensity of the PSD function with oblique incidence for q < q_0_ reveal long-range smoothening of the sputtered surfaces, as inferred from the roughness measurements. Thus, the underlying mechanism relies on the surface confined viscous flow i.e. ion beam stimulated mass rearrangement inside the amorphous layers^35–37^.

Further, to quantify the role of oblique incidence induced sputtering phenomena in the observed surface dynamics, we have calculated the sputtering yield, energy transferred to recoil and projected range using SRIM simulations^38^ for the oblique incidences of 30°, 40° and 50°. Table 3 lists the values of these parameters for different oblique incidences.

**Table 3:** Values of sputtering yield, energy transferred to recoils and projected range for argon ions for 30^0^, 40^0^ and 50^0^ oblique incidence.

80 keV Ar^+^ on Si (111) at oblique incidence of	Sputtering Yield (Si atoms/Ar^+^ ion)	Energy transferred to recoils	Projected Range of Ar (nm)
30°	0.96	70	77 ± 31
40^0^	1.35	80	65 ± 30
50^0^	2.16	100	55 ± 28

It is quite clear from the Table 3 that the sputtering yield (Si atoms/Ar^+^ ion) and energy transferred to recoiling Si atoms is highest for 50^0^ off-normal incidence while range of incident argon ions is lowest. This shows that for oblique incidences of 50^0^, the incident argon ions majorly impart their energy to the silicon surface atoms. As a result, more surface atoms achieve enough energy to overcome the surface binding energy and leave the surface thus, initiating the sputtering process^36,37,39,40^. This, in turn, produces point defects and accumulation of damage in the surface region of Si lattice. So, small crystalline content in the vicinity of amorphous phase is present in modified surface layers for this oblique incidence. With decrease in oblique incidence to 40^0^, the sputtering yield and energy transferred to recoils decreases. Thus, this energy deposition instead of being only on the surface extends to deeper layers and as a result, the damage distribution now extends from the surface to near surface region. The stable structure of silicon also starts breaking and Si-Si bond angle starts deviating from normal tetrahedral angle.

But with further decrease in oblique incidence to 30^0^, the energy deposition of incident ions extends further to near surface region leading to instability in the Si lattice with breaking of bonds and finally collapses to the amorphous phase. Hence crystalline to amorphous phase transformation occurs. This structural phase transition is generally accompanied by line-width narrowing of the 1-TO peak (see Table 2) which could be due to the production of point defects and stress leading to change of lattice bond angle. Hence, crystalline to amorphous phase transition is observed at lowest oblique incidence of 30^0^.

Interestingly, this angle dependent energy deposition by incoming argon ions accounts for the observed decrease in the size of evolved defects and corresponding increase in the defect number density. For 50^0^ off-normal incidence, the projected range of incoming argon ions is 55 ± 28 nm (Table 3). This low projected range resulted in energy deposition only in surface region and thus initiating the sputtering of Si atoms lying in this surface region. Hence, defects of bigger size and height are formed at this oblique incidence.

With decrease in oblique incidence to 40^0^, the projected range of these incident argon ions increases to 65 ± 30 nm (Table 3). This increase in penetration depth results in energy deposition of the argon ion in both surface and near surface region. Thus, size and height of evolved defects decreases. With further decrease in oblique incidence to 30^0^, the range increase to 77 ± 31 nm (Table 3). As a consequence, the major portion of this energy deposition is now in the near surface region as compared to surface region. This decline in energy deposition in the surface layers accounts for the observed decrease in the size and height of evolved defects at this oblique incidence.

It is worth mentioning that defect density is related to the size of the nano-dimensional defects. So, evolution of bigger defects at oblique incidence of 50^0^ results in lower defect density of 4.5 × 10^8^ cm^−2^. As the size of these structural defects decreases with decrease in off-normal incidence, so, increase in defect number density has been observed.

## Conclusions

Our findings provide a reliable way for controlled surface modification of Si(111) surfaces under oblique ion beam irradiation. Si(111) surfaces exhibit densely packed and self-assembled nano-sized defect structures having distinct dependence on the oblique incidence of incident argon ions. The nanosized defects exhibit fairly homogenous distribution with average size of 87 to 42 nm and height of 12 to 5 nm respectively. Interestingly, larger defects zones have been observed to be comprising of several nanosized defects. The crystalline to amorphous phase (c/a) transition under Ar^+^ ion sputtering has been observed. The decrease in the surface roughness is related to the smoothening of the surface via relaxed stress. Raman scattering reveal that c-Si and a-Si peaks shift towards lower wavenumber followed by line-width narrowing with respect to decrease in oblique incidence. The decreased strain due to softening of LO and TO modes in the irradiated specimens leads to smoothening of the surfaces. We believe that formation of such defect features relies on ion induced viscous flow process i.e. ion beam induced mass rearrangement from the c/a interface towards the surface. The present work provides a route for the defect engineering in technologically important group IV materials, key substrates in nano-applications starting from integrated circuits and expanding into microelectronics.

## References

[1] Kinchin GW, Pease RS (1955). The displacement of atoms in solids by radiation. Rep. Prog. Phys..

[2] Krasheninnikov AV, Nordlund K (2010). Ion and electron irradiation-induced effects in nanostructured materials. J. Appl. Phys..

[3] Carter, G. Nobes, M. J., Katardjiev, I. V. & Whitton, J. L. Defect and diffusion forum, in *Ion Implantation*. 97. Ed. Trans. Techn. Publ. Ltd. (1988).

[4] Paramanik D, Majumder S, Sahoo SR, Varma S (2013). Nano Pattern Formation and Surface Modifications by Ion Irradiation. Defence Science Journal.

[5] Winterbon, K. B. *Ion Implantation Range and Energy Deposition Distributions*. Plenum, New York, (1975).

[6] Jiang W (2009). Response of nanocrystalline 3C silicon carbide to heavy-ion irradiation. Phys. Rev. B: Condens. Matter Mater. Phys..

[7] Newman RC (1982). Defects in Silicon. Rep. Prog. Phys..

[8] Dimaria DJ, Kerr DR (1975). Interface effects and high conductivity in oxides grown from polycrystalline silicon. Appl. Phys. Lett..

[9] Dey S, Roy C, Pradhan A, Varma S (2000). Raman scattering characterization of Si(100) implanted with mega-electron-volt Sb. J. Appl. Phys..

[10] Jagadish C, Svensson BG, Hauser N (1993). Point defects in n-type silicon implanted with low doses of MeV boron and silicon ions. Semicond. Sci. Technol..

[11] Wallace JB, Bayu Aji LB, Shao L, Kucheyev SO (2019). Impact of pre-existing disorder on radiation defect dynamics in Si. Sci. Rep..

[12] Pastuovic Z (2014). Generation of vacancy cluster-related defects during single MeV silicon ion implantation of silicon. Nucl. Instruments Methods Phys. Res. Sect. B Beam Interact. Mater. Atoms..

[13] Dey S, Pradhan A, Varma S (2000). Damage studies of MeV Sb-implanted Si(100) by channeling and Raman spectroscopy. Journal of Vacuum Science & Technology B: Microelectronics and Nanometer Structures Processing, Measurement, and Phenomena.

[14] Wong-Leung J, Jagadish C, Conway MJ, Fitz Gerald JD (2001). Effect of implant temperature on secondary defects created by MeV Sn implantation in silicon. J. Appl. Phys..

[15] Facsko S (1999). Formation of Ordered Nanoscale Semiconductor Dots by Ion Sputtering. Science.

[16] Kumar T, Khan SA, Singh UB, Verma S, Kanjilal D (2012). Formation of nanodots on GaAs by 50 keV Ar+ ion irradiation. Appl. Surf. Sci..

[17] Eklund, E. A., Snyder, E. J. & Williams, R. S. Submicron-scale surface roughening induced by ion bombardment. *Phys*. *Rev*. *Lett*. **67** (1991). 10.1103/PhysRevLett.67.1759 10044240

[18] Som T, Chini TK, Katharia YS, Tripathy S, Kanjilal D (2009). Formation of nanodots on oblique ion sputtered InP surfaces. Appl. Surf. Sci..

[19] Gago R, Vazquez L, Cuerno R (2001). Production of ordered silicon nanocrystals by low-energy ion sputtering. Appl. Phys. Lett..

[20] Jadan M, Chelyadinskii AR, Yavid V (2004). Yu., Efficiency of formation of radiation defects in silicon upon implantation of silicon and phosphorus ions. Nucl. Instrum. Methods Phys. Res. B.

[21] Zhao, Y. P., Wang, G. C. & Lu, T. M. Characterization of Amorphous and Crystalline Rough Surfaces: Principles and Applications (Academic Press, San Diego 2001).

[22] Eklund EA, Snyder EJ, Williams RS (1993). Correlation from randomness: quantitative analysis of ion-etched graphite surfaces using the scanning tunneling microscope. Surface Science.

[23] Gibbons JF (1972). Ion implantation in semiconductors—Part II: Damage production and annealing. Proc. IEEE.

[24] Herre O (1998). Formation of discontinuous tracks in single-crystalline InP by 250-MeV Xe-ion irradiation. Phy. Rev. B..

[25] Sahu G (2014). Confinement in MeV Au2+ implanted Si: a Raman scattering study. Adv. Nat. Sci.: Nanosci. Nanotechnol..

[26] Watkins, G. D. *Radiation Effects in Semiconductors*, 67–81. Ed. Plenum, New York (1968).

[27] Beeman D, Tsu R, Thorpe MF (1985). Structural information from the Raman spectrum of amorphous silicon. Phy. Rev..

[28] Nita N, Schaeublin R, Victoria M, Valiev RZ (2005). Effects of irradiation on the microstructure and mechanical properties of nanostructured materials. Philos. Mag..

[29] Mishra P (2014). Structural and optical study of MeV cobalt ion implanted silicon. Adv. Mat. Lett..

[30] Lavrentiev V, Vacik J, Vorlicek V, Vosecek V (2010). Raman scattering in silicon disordered by gold ion implantation. Phys. Status Solidi B.

[31] Smith JE, Brodsky MH, Crowder BL, Nathan ML (1971). Raman Spectra of Amorphous Si and Related Tetrahedrally Bonded Semiconductors. Phys. Rev. Lett..

[32] Zwick A, Carles R (1993). Multiple-order Raman scattering in crystalline and amorphous silicon. Phys. Rev. B.

[33] Petri R (1994). Silicon roughness induced by plasma etching. J. Appl. Phys..

[34] Siva V, Datta DP, Singh A, Som T, Sahoo PK (2016). Nanocomposite synthesis and photoluminescence properties of MeV Au-ion beam modified Ni thin films. Appl. Surf. Sci..

[35] Kumar T (2012). Role of surface composition in morphological evolution of GaAs nano-dots with low-energy ion irradiation. Nano. Res. Lett..

[36] Sigmund P (1969). Theory of Sputtering. I. Sputtering Yield of Amorphous and Polycrystalline Targets. Phys. Rev..

[37] Carter G (2001). The physics and applications of ion beam erosion. J. Phys. D: Appl. Phys..

[38] Zeigler, M. D. & Biersack, J. P. SRIM 2008.04 software, http://www.srim.org (2008).

[39] Sigmund, P. Sputtering by Ion Bombardment, Theoretical Concepts. In *Sputtering by Particle Bombardment 1*. *Topics in Applied Physics* 47, 9. Ed. Springer-Verlag, Berlin, (1981).

[40] Chan WL, & E, Chason E (2007). Making waves: Kinetic processes controlling surface evolution during low energy ion sputtering. J. Appl. Phys..

